# Transpedicular bone grafting and pedicle screw fixation in injured vertebrae using a paraspinal approach for thoracolumbar fractures: a retrospective study

**DOI:** 10.1186/s13018-016-0452-4

**Published:** 2016-10-17

**Authors:** Qinliang Li, Cai Yun, Shichun Li

**Affiliations:** Department of Orthopaedic, Shijingshan Teaching Hospital of Capital Medical University, Shijingshan Hospital of Beijing City, Beijing, 100043 China

**Keywords:** Thoracolumbar fractures, Injured vertebrae, Bone graft, Fixation, Paraspinal approach

## Abstract

**Background:**

Complications in posterior pedicle screw fixation using a conventional posterior approach for thoracolumbar fractures include vertebral height loss, kyphosis relapse and breakage, or loosening of instrumentation. The purpose of this study was to evaluate the clinical effects of transpedicular bone grafting and pedicle screw fixation in injured vertebrae using a paraspinal approach for thoracolumbar fractures.

**Methods:**

We retrospectively analyzed 50 patients with thoracolumbar fractures treated with transpedicular bone grafting and pedicle screw fixation in injured vertebrae using a paraspinal approach. Operative time, blood loss, visual analog scale (VAS) scores for back pain, and the relative height and Cobb angle of the fractured vertebrae were measured.

**Results:**

The average operative time was 71.8 min, and the blood loss was 155 ml. Postoperative VAS scores were significantly lower than preoperative scores (*P* = 0.08), but there was no difference between 1 week and 1 year postoperatively (*P* = 0.18). The postoperative relative heights of the fractured vertebrae were higher than the preoperative heights (*P* = 0.001, 0.005, 0.001), but there were no differences between 1 week and 1 or 2 years postoperatively (*P* = 0.24/0.16). The postoperative Cobb angles were larger than the preoperative angles (*P* = 0.002, 0.007, 0.001), but there were no differences between 1 week and 1 or 2 years postoperatively (*P* = 0.19/0.23).

**Conclusions:**

Transpedicular bone grafting and pedicle screw fixation in injured vertebrae using a paraspinal approach for thoracolumbar fractures achieved satisfactory results and can restore vertebral height, increase the stability of the anterior and middle columns of injured vertebrae, and decrease the risk of back pain.

## Background

Posterior pedicle screw fixation using a conventional posterior approach is widely used for thoracolumbar fractures [1, 2] and includes pedicle screw fixation one vertebra above and one vertebra below the fracture. However, the incidence of vertebral height loss, kyphosis relapse and breakage, or loosening of instrumentation is high [3, 4]. Moreover, degeneration of the back muscles was found in patients who underwent a conventional posterior approach [5, 6], and most patients had persistent back pain [7]. Recently, studies have shown that screw implantation in the injured vertebra exhibits stronger fixation, and more effectively prevents loosening or breakage of instrumentation, compared with traditional four-screw cross-segmental fixation [8, 9]. Transpedicular intracorporeal bone grafting has been used to promote vertebral fracture healing and maintain vertebral height [10, 11]. A paraspinal approach has been used for thoracolumbar fractures in order to decrease the damage to back muscles and the risk of postoperative back pain [12, 13]. On the basis of the preceding information, we conducted a retrospective study on 50 patients with thoracolumbar fractures without neurological deficits treated with short-segment fixation combined with transpedicular bone grafting and pedicle screw fixation in injured vertebrae using a paraspinal approach; these cases achieved satisfactory clinical results.

## Methods

### General data

The 50 cases with single-level thoracolumbar fractures comprised 36 males and 14 females, with an average age of 46 years (range 28–63 years). This study was conducted in accordance with the Declaration of Helsinki. This study was conducted with approval from the Ethics Committee of Beijing Shijingshan Hospital. Written informed consent was obtained from all participants. The fractures were caused by falling from a height (*n* = 16), road accident (*n* = 24), collision with a heavy object (*n* = 6), and falling down (*n* = 4) and involved single segment vertebral fractures: T10 in 2 cases, T11 in 7, T12 in 16, L1 in 13, and L2 in 12. The fractures were classified according to Denis criteria [14]: 28 cases of burst fracture and 22 of flexion-compression fracture; all cases were type A according to AO criteria [14]. All thoracolumbar injury classification and severity scores (TLICS) were above 4 [15]. Transpedicular computed tomography (CT) was performed to evaluate fractured vertebrae: 16 patients had unilateral intact pedicles and 34 had bilateral intact pedicles. No patients had a neurological deficit, severe osteoporosis (dual-energy X-ray absorptiometry (DEXA) score >2.5 T-score), or morbid obesity (>127 kg). The time from injury to operation was less than 1 week. The patients were treated with posterior short-segment fixation combined with transpedicular bone grafting and pedicle screw fixation in injured vertebrae using a paraspinal approach, including 34 cases with bilateral and 16 with unilateral screw implantation.

### Surgical procedures

All procedures were performed under controlled general anesthesia with endotracheal intubation in the prone position. A midline skin incision was made, and subcutaneous flaps were elevated bilaterally. A fascial incision was made approximately 1.5–2.5 cm lateral to the spinous process. We bluntly dissected between the multifidus and longissimus muscles using a finger and placed self-retaining retractors between the two muscle groups, then exposed part of the facet joint. The pedicle screws were implanted in the injured vertebrae and the upper and lower vertebrae. Fixation was achieved through connecting rods, producing distraction, compression, and slight lordosis. One of the screws in the injured vertebra was removed, followed by intravertebral bone grafting. The iliac bone was reduced to particles and injected into the vertebra through a sheath and pushrod; then, the removed screw was implanted and the rod was connected. No direct decompression or bone fusion was performed, and no incision drainage was used in any of the patients.

### Postoperative management

Ambulatory activities while wearing a brace were encouraged within 3 days postoperatively, and the brace was retained for 8 weeks. Strenuous labor and sports activity were prohibited for 3 months. The pedicle screws were removed 2 years postoperatively.

### Measurement and observation

The operative time and blood loss were recorded. Pre- and postoperative VAS scores for back pain were obtained over a minimum 1-year follow-up period.

**Fig. 1:**
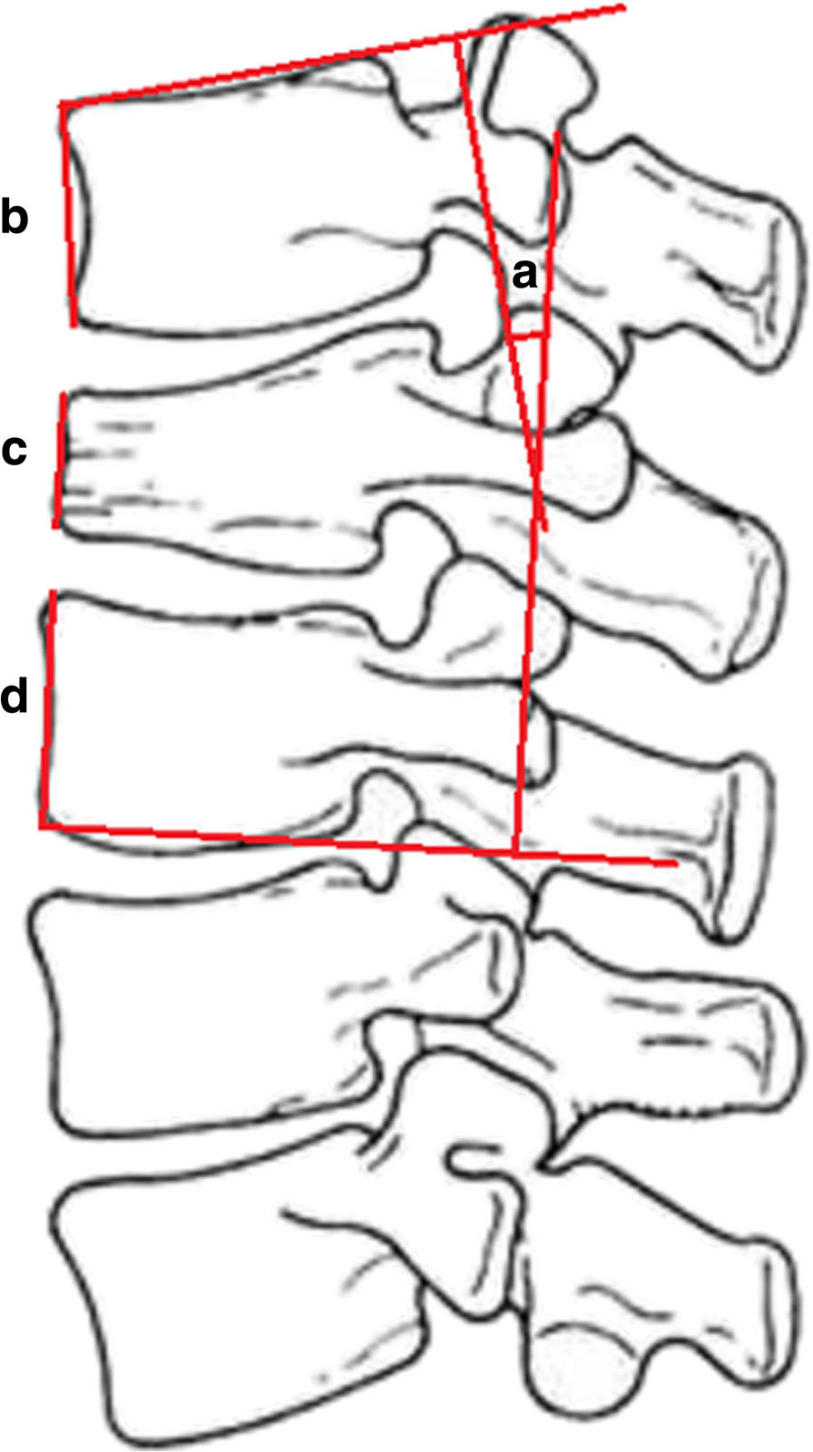
**a** Cobb angle. **b** Height of the anterior fractured vertebra. **c** Height of the upper anterior vertebra. **d** Height of the lower anterior vertebra

Anterior vertebral height represented the height of the fractured vertebra (Fig. 1(b)), and the mean value of the upper and lower vertebral heights represented the normal height of the fractured vertebra (Fig. 1(c, d)); the relative height of the fractured vertebra was the percentage of height of the fractured vertebra relative to that of the normal height of the fractured vertebra.

The angle between the upper terminal plate of the upper vertebra and the lower terminal plate of the lower vertebra is shown in Fig. 1(a).

### Statistical analysis

Data are expressed as the mean ± standard deviation and analyzed by one-way analysis of variance and multiple comparison methods with SPSS software, version 16.0 (SPSS Inc., Chicago, IL, USA). *P* < 0.05 was considered to indicate a statistically significant difference.

## Results

### Operative time and blood loss

The median operative time was 71.8 min (range 58–92 min), and the median blood loss was 155 ml (range 95–210 ml).

### Pre- and postoperative VAS

No patient had persistent postoperative back pain. Postoperative VAS scores at 1 week (3.66) and at 1 year (3.54) were significantly lower than the preoperative scores (8.55 ± 0.76) (*P* = 0.008, 0.011). VAS scores decreased over time, but there was no significant difference between 1 week and 1 year postoperatively (*P* = 1.15).

### Relative height of fractured vertebra

**Fig. 2:**
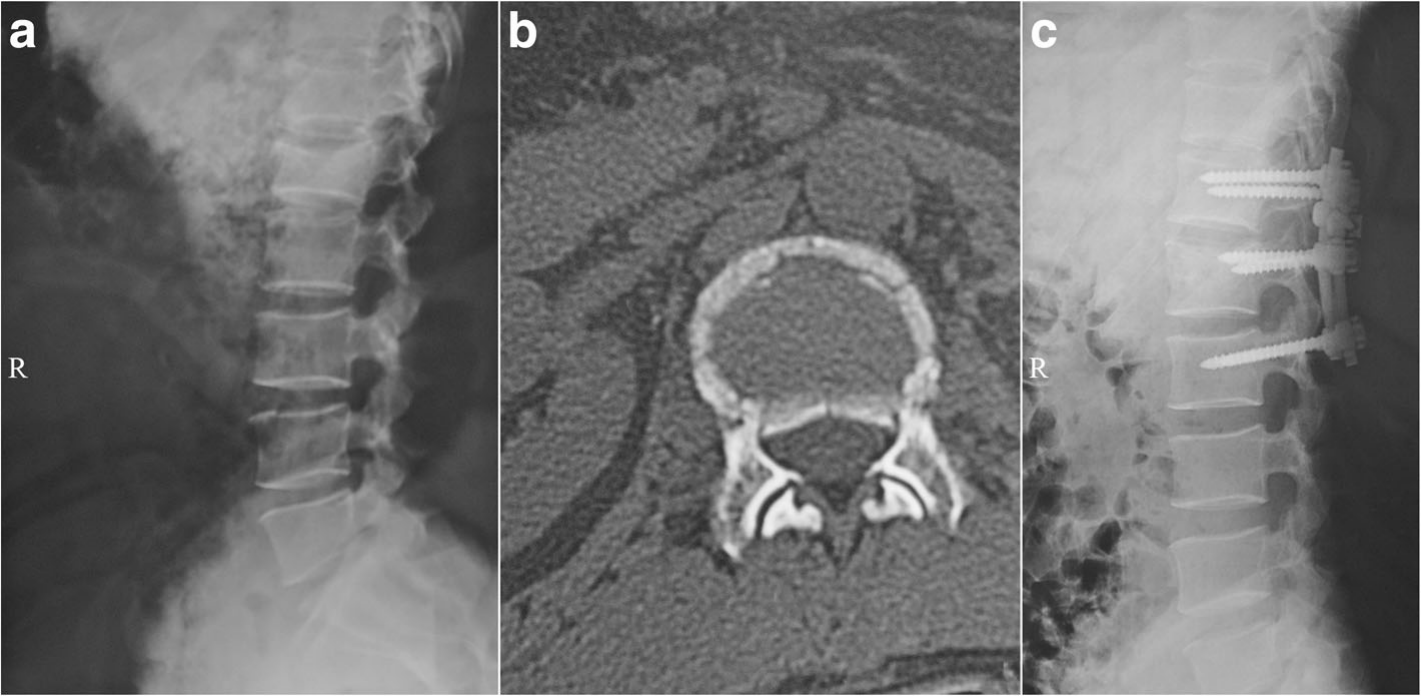
**a** Preoperative lateral radiograph of a 35-year-old female patient with a compression fracture at L1. **b** Preoperative CT of the patient. **c** Lateral postoperative radiograph of the patient

**Fig. 3:**
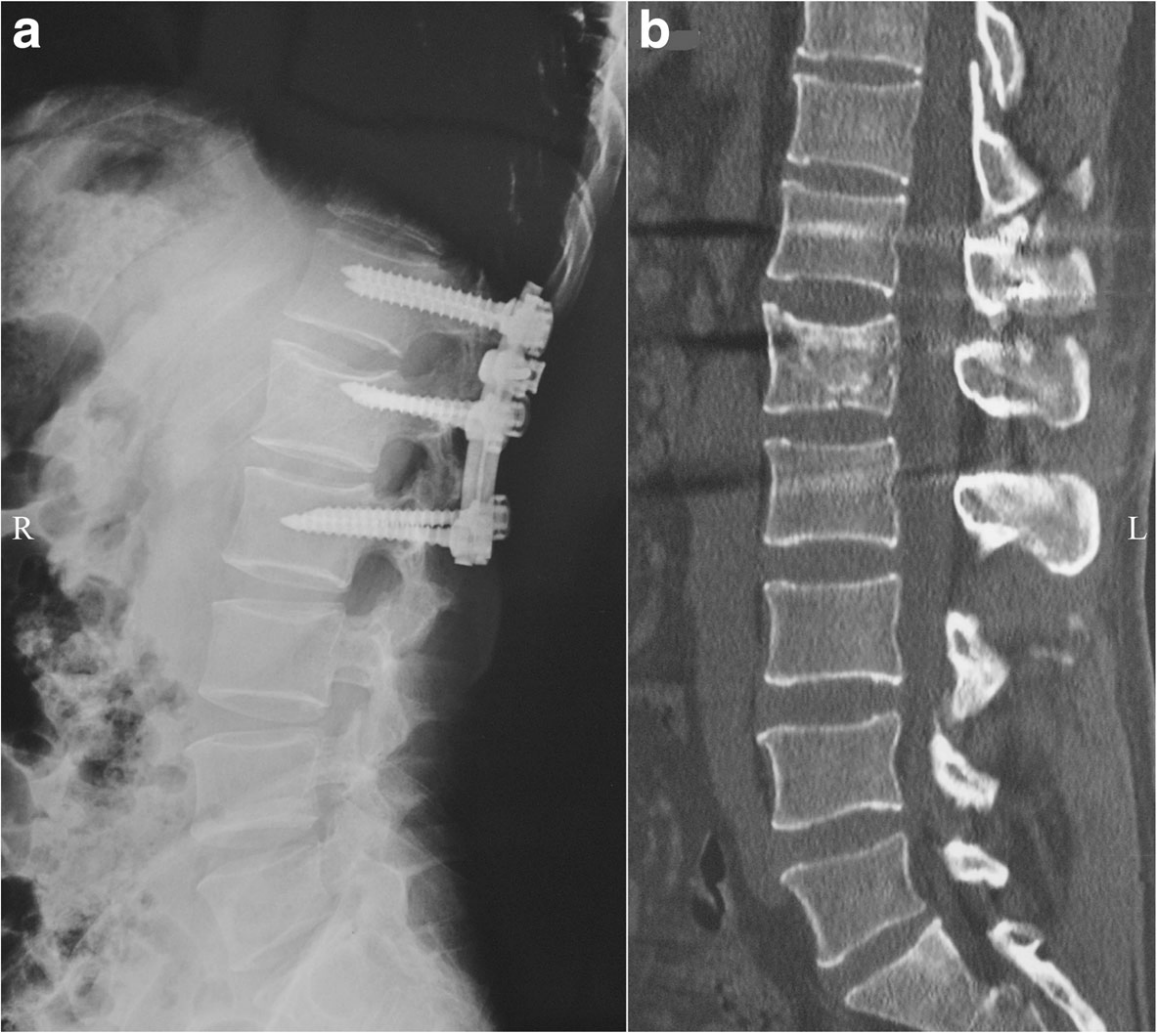
**a** Lateral radiograph of the patient shown in Fig. 2 at 1 year postoperatively. **b** CT of the patient shown in Fig. 2 at 1 year postoperatively

**Fig. 4:**
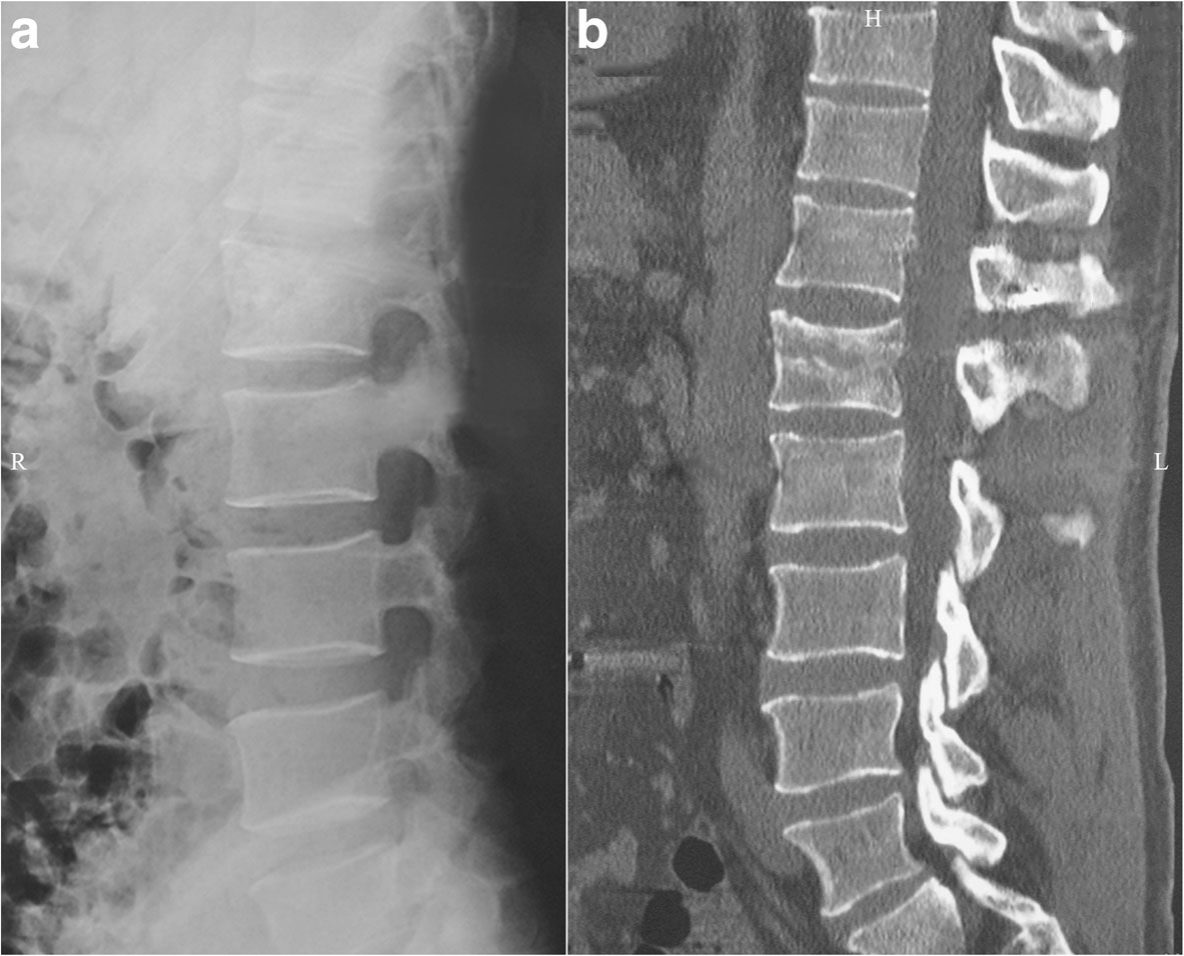
**a** Lateral radiograph of the patient shown in Fig. 2 at 2 years postoperatively. **b** CT of the patient shown in Fig. 2 at 2 years postoperatively

CT and X-ray reexamination showed that the bone graft was well seated in the injured vertebra, with no screw loosening, breakage, or rod breakage (Figs. 2, 3, and 4). The relative height of the fractured vertebra was 56.8 % preoperatively, 91.2 % 1 week postoperatively, 89.6 % 1 year postoperatively, and 88.7 % 2 years postoperatively. The height of the fractured vertebra was significantly restored postoperatively (*P* = 0.001, 0.005, and 0.001, Table 1). There were no differences between 1 week and 1 year postoperatively (*P* = 0.24) or between 1 week and 2 years postoperatively (*P* = 0.16).

**Table 1:** Relative heights of the fractured vertebrae and Cobb angles

Item	Number	Preoperative	1 week postoperatively	1 year postoperatively	2 years postoperatively
Relative height of fractured vertebrae (%)	50	56.8 ± 15.6	91.2 ± 10.7^a^	89.6 ± 10.3^b^	88.7 ± 9.6^c^
Cobb angle (°)	50	18.2 ± 2.6	7.5 ± 1.4^d^	8.7 ± 1.1^e^	8.2 ± 1.5^f^

^a^ *P* = 0.001; ^b^ *P* ≤ 0.005; ^c^ *P* = 0.001; ^ab^ *P* = 0.24; ^bc^ *P* = 0.16; ^d^ *P* = 0.002; ^e^ *P* ≤ 0.007; ^f^ *P* = 0.001; ^de^ *P* = 0.19; ^ef^ *P* = 0.23

### Cobb angle

The mean Cobb angle was 18.2° preoperatively, 7.5° 1 week postoperatively, 8.7° 1 year postoperatively, and 8.2° 2 years postoperatively. The Cobb angle was significantly restored postoperatively (*P* = 0.002, 0.007, 0.001). There were no differences between 1 week and 1 year postoperatively (*P* = 0.19, Table 1) or between 1 week and 2 years postoperatively (*P* = 0.23, Table 1).

## Discussion

Posterior fixation has become a popular method for the treatment of thoracolumbar burst and compression fractures. For a single-level vertebral fracture, four-screw pedicle fixation has been used in the respective upper and lower vertebrae [1, 2, 16, 17]; however, this cannot achieve satisfactory reduction in the absence of ligament and annulus fibrosus traction. Moreover, the stress concentrated at the pedicle screws leads to a high incidence of loosening and breakage of fixation and loss of vertebral height or kyphosis [3, 4, 18, 19]. In order to reduce the incidence of these complications, the screws were fixed to the injured vertebra [8, 9, 20, 21]; this did not increase fixation length, and it maintained motion segments of the spine as much as possible, dispersed load bearing, supported the spine, and maintained reduction until bony fusion. The screws implanted in the injured vertebra created a supporting point on the fractured vertebra, which helped to rearrange and reduce the adjacent bone blocks [22]. In addition, screws in the injured vertebra can correct kyphosis or horizontal displacement, share stress from other internal fixation during spinal lateral flexion or rotation, and reduce the likelihood of loosening and breakage of fixation.

In our study, we retrospectively analyzed 50 patients with thoracolumbar fractures treated with pedicle screw fixation in injured vertebrae. According to the integrity of the pedicle, unilateral or bilateral pedicle screw implantation was performed. The vertebral height was maintained and the Cobb angle was corrected. No screw loosening or breakage or rod breakage was observed.

A large space following vertebral reduction influenced instability of the spinal anterior central column and ultimately resulted in loss of vertebral height [23]. Some studies attempted to infuse bone cement into the injured vertebra to fill the space, and achieved some clinical efficacy [24]. However, there were some potential risks: the long-term presence of bone cement in young patients can produce cutting effects. Previous studies discussed the importance of bone grafting in the fractured vertebra, which can restore the stability of the collapsed anterior and central column [10, 23, 25]. Our study performed transpedicular bone grafting. CT results at 1 year of follow-up showed good fracture healing in all patients. The vertebral height was maintained. Filling with bone in the anterior and middle columns resulted in better correction and stronger support for reconstruction because of the intravertebral pressure, and correction loss was minimized.

Degeneration of the back muscle was found in patients who underwent posterior surgery, and most patients had persistent back pain [7]. The postoperative muscle abnormalities are believed to be related to the pressure and duration of retraction of the muscles during surgery [5–7] and may be due to denervation [26]. In recent years, the paraspinal approach has been widely used for thoracolumbar fractures in order to decrease the damage to the back muscle and the risks of postoperative back pain [12, 13, 27, 28]. The paraspinal approach to lumbar spine surgery goes between the lateral border of the sacrospinalis muscles and the quadratus lumborum muscle, and the original use was for spinal fusion [27, 29, 30]. With this approach, a one-level or multilevel fusion could be performed. In Wiltse’s study, the fascial incision was made only 2 cm lateral to the midline [27, 29]. In our present study, the distances of the fascial incision to the midline were about 1.5 cm at T10; 2 cm at T11, T12, and L1; and 2.5 cm at L2 and were similar to those of another study [27]. The approach was simple, allowed access to the articular and transverse processes, and had great advantage in thoracolumbar vertebral fracture surgery without decompression. Without wide muscular disinsertion, the approach left the supraspinous and interspinous ligaments intact; without denervation of the back muscle, it showed obvious advantages in operative time and blood loss and decreased the incidence of postoperative back pain. In our study, the operative time was 71.8 min on average, the blood loss was 155 ml on average, and no patient had persistent postoperative back pain; VAS scores for back pain postoperatively were significantly lower than the preoperative scores.

Inclusion criteria for transpedicular intravertebral bone grafting and pedicle screw fixation in injured vertebrae using a paraspinal approach for thoracolumbar fractures were as follows: (1) type A according to AO criteria; (2) less than 50 % narrowing of the spinal canal by the vertebral fragment and less than 90° retroflexion of the vertebral fragment; (3) unilateral or bilateral integrity of the pedicle of the injured vertebra; (4) fresh fracture, less than 1 week from injury; and (5) neurological status D or E degree according to Frankel. Exclusion criteria were morbid obesity (>127 kg) and severe osteoporosis (DEXA score <2.5 T-score) [29].

## Conclusions

Transpedicular intravertebral bone grafting and pedicle screw fixation in injured vertebrae using a paraspinal approach achieved favorable clinical results for thoracolumbar fractures. This method restored vertebral height, increased anterior and central column stability, and reduced fixation loosening or breakage. Furthermore, it is less invasive, with reduced blood loss, and decreases the risk of postoperative back pain. However, this technique has only been used for treatment of thoracolumbar fractures for a short period of time, and the number of cases and duration of follow-up has been limited. Further follow-up is needed to verify long-term efficacy.

## Abbreviations

CT: Computed tomography
TLICS: Thoracolumbar injury classification and severity score
VAS: Visual analog scales
